# Association Between Stiffness of the Deep Fibres of the Tibialis Anterior Muscle and *Seiza* Posture Performance After Ankle Fracture Surgery

**DOI:** 10.3390/jfmk10030300

**Published:** 2025-08-01

**Authors:** Hayato Miyasaka, Bungo Ebihara, Takashi Fukaya, Koichi Iwai, Shigeki Kubota, Hirotaka Mutsuzaki

**Affiliations:** 1 Department of Rehabilitation, Tsuchiura Kyodo General Hospital, 4-1-1 Otsuno, Tsuchiura 300-0028, Ibaraki, Japan; miyasaka1853h@yahoo.co.jp (H.M.); bun.hirakata@gmail.com (B.E.); 2 Graduate School of Health Sciences, Ibaraki Prefectural University of Health Sciences, 4669-2 Ami, Ami 300-0394, Ibaraki, Japan; 3 Department of Physical Therapy, Faculty of Health Sciences, Tsukuba International University, 6-8-33 Manabe, Tsuchiura 300-0051, Ibaraki, Japan; t-fukaya@tius.ac.jp; 4 Center for Humanities and Sciences, Ibaraki Prefectural University of Health Sciences, 4669-2 Ami, Ami 300-0394, Ibaraki, Japan; iwai@ipu.ac.jp; 5 Department of Occupational Therapy, School of Health Sciences, Ibaraki Prefectural University of Health Sciences, 4669-2 Ami, Ami 300-0394, Ibaraki, Japan; kubotashi@ipu.ac.jp; 6 Center for Medical Science, Ibaraki Prefectural University of Health Sciences, 4669-2 Ami, Ami 300-0394, Ibaraki, Japan; 7 Department of Orthopedic Surgery, Ibaraki Prefectural University of Health Sciences Hospital, 4773 Ami, Ami 300-0331, Ibaraki, Japan

**Keywords:** tibialis anterior, stiffness, elastography, shear modulus, sitting posture

## Abstract

**Background:** *Seiza*, a traditional sitting posture requiring deep ankle plantarflexion and knee flexion, often becomes difficult after ankle fracture surgery because of restricted mobility. Increased stiffness of the tibialis anterior (TA) muscle, particularly in its deep and superficial fibres, may limit plantarflexion and affect functional recovery. This study aimed to investigate the relationship between TA muscle stiffness, assessed using shear wave elastography (SWE), and the ability to assume the seiza posture after ankle fracture surgery. We also sought to determine whether the stiffness in the deep or superficial TA fibres was more strongly correlated with seiza ability. **Methods:** In this cross-sectional study, 38 patients who underwent open reduction and internal fixation for ankle fractures were evaluated 3 months postoperatively. Seiza ability was assessed using the ankle plantarflexion angle and heel–buttock distance. The shear moduli of the superficial and deep TA fibres were measured using SWE. Ankle range of motion, muscle strength, and self-reported seiza pain were also measured. Multiple linear regression was used to identify the predictors of seiza performance. **Results:** The shear moduli of both deep (β = −0.454, *p* < 0.001) and superficial (β = −0.339, *p* = 0.017) TA fibres independently predicted ankle plantarflexion angle during seiza (adjusted R^2^, 0.624). Pain during seiza was significantly associated with reduced plantarflexion, whereas muscle strength was not a significant predictor. **Conclusions:** TA muscle stiffness, especially in the deep fibres, was significantly associated with limited postoperative seiza performance. Targeted interventions that reduce deep TA stiffness may enhance functional outcomes.

## 1. Introduction

*Seiza* is a traditional sitting posture widely used in Asian and Middle Eastern cultures in daily life, religious practices, and martial arts such as judo and kendo [1,2,3,4]. This posture requires a considerable range of motion (ROM), involving approximately 150° of knee flexion and 60° of ankle plantarflexion, to achieve full contact between the buttocks and heels [5,6]. Consequently, numerous studies have investigated the ability of patients to adopt this posture after knee joint surgery [4,5,7,8]. However, few studies have investigated seiza ability after ankle joint surgery [9]. Among various ankle surgeries, those for ankle fractures are particularly common and are projected to increase in frequency [6,10,11,12], making them an important area of clinical focus. Following surgical treatment of ankle fractures, restrictions in ankle plantarflexion ROM are frequently observed and are believed to hinder the ability to assume the seiza position [13]. One potential contributing factor is the stiffness of the tibialis anterior (TA) muscle, a major dorsiflexor with a large physiological cross-sectional area, which may restrict the required plantarflexion [14]. However, the precise relationship between TA muscle stiffness and seiza posture remains unclear. Considering that the TA is a pennate muscle composed of superficial and deep fibre regions [15,16,17], the region that contributes most to the muscle’s overall stiffness should be clarified.

Shear wave elastography (SWE) is a non-invasive, quantitative, and localised method for measuring the stiffness of muscles and tendons by calculating their shear modulus [18,19]. Previous studies have demonstrated the utility of SWE in assessing muscle stiffness, including that of the TA muscle [20]. These studies suggest that SWE provides important insights into muscle function, such as detecting changes in stiffness related to muscle-tendon length and force, as well as potentially capturing functionally relevant adaptations over time. By assessing the shear modulus of the TA muscle, SWE could help clarify the relationship between TA muscle stiffness and biomechanical factors, such as heel–buttock distance and ankle plantarflexion angle during seiza. Moreover, even in individuals without a history of ankle trauma or surgery, naturally increased stiffness of the TA muscle resulting from habitual muscle tightness, postural tendencies, or limited flexibility, may influence the ability to assume the seiza posture.

This cross-sectional observational study aimed to investigate whether the shear modulus of the TA muscle is associated with the ability to sit in the seiza position, as reflected by heel–buttock distance and ankle plantarflexion angle, in patients after ankle fracture surgery. In addition, this study sought to identify the specific region of the TA muscle that is more strongly associated with the ability to sit in seiza. We hypothesised that the deep fibres of the TA muscle would be more strongly associated with seiza ability based on their anatomical positioning and presumed a greater contribution to passive resistance during plantarflexion. If these hypotheses are supported, these findings could inform rehabilitation strategies aimed at improving ankle mobility and facilitating the return to functional activities, including sitting in seiza, after ankle fracture surgery. Specifically, targeting deep TA stiffness could provide more effective therapeutic strategies aimed at restoring full plantarflexion required for traditional floor-sitting postures in populations where cultural activities necessitate mobility.

## 2. Materials and Methods

### 2.1. Participants

**Figure 1:**
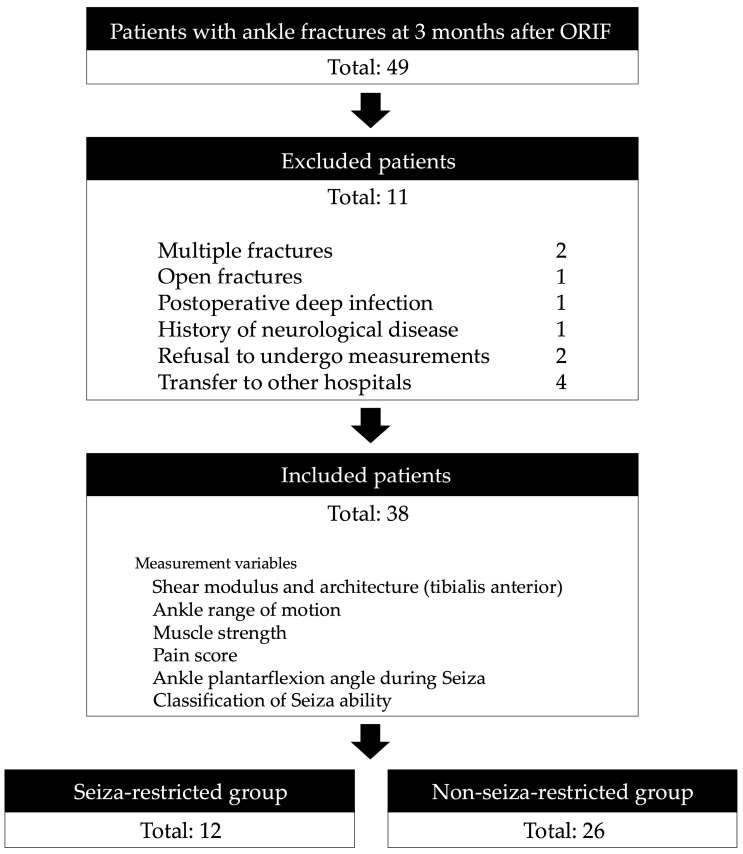
Flowchart of participant inclusion. Participants were categorised into two groups based on their ability to sit in the seiza posture. Participants who were unable to sit in the seiza posture due to physical limitations were categorised as the seiza-restricted group, whereas those who could maintain the posture were categorised as the non-seiza-restricted group.

This cross-sectional observational study was conducted at a single hospital between July 2022 and February 2025 and included patients with ankle fractures who were admitted to our hospital. Patients diagnosed with unimalleolar, bimalleolar, or trimalleolar ankle fractures based on radiographic imaging, who underwent open reduction and internal fixation (ORIF) followed by physical therapy, were included. All patients underwent ORIF and were immobilised in a splint for at least 1 week postoperatively. Those with multiple fractures, open fractures, postoperative complications such as deep infection or deep vein thrombosis, or a history of neurological or orthopaedic diseases and those who had already completed follow-up visits at our hospital prior to the study period were excluded. All assessments were uniformly performed 3 months post-ORIF for all participants. All participants used crutches for at least the first 3 weeks and continued a rehabilitation programme focusing on joint mobility, muscle strength, and functional activities, such as walking and stair climbing. Demographic and clinical information, including age, sex, height, number of fractures [21,22], and Lauge-Hansen classification [23], were obtained from medical records. Body weight was measured using a digital scale, and body mass index (BMI) was calculated accordingly. Participants were categorised into two groups based on their ability to sit in the seiza posture: the seiza-restricted group and the non-seiza-restricted group (Figure 1).

### 2.2. Measurement of the Ankle Plantarflexion Angle During Seiza and Classification of Seiza Performance

**Figure 2:**
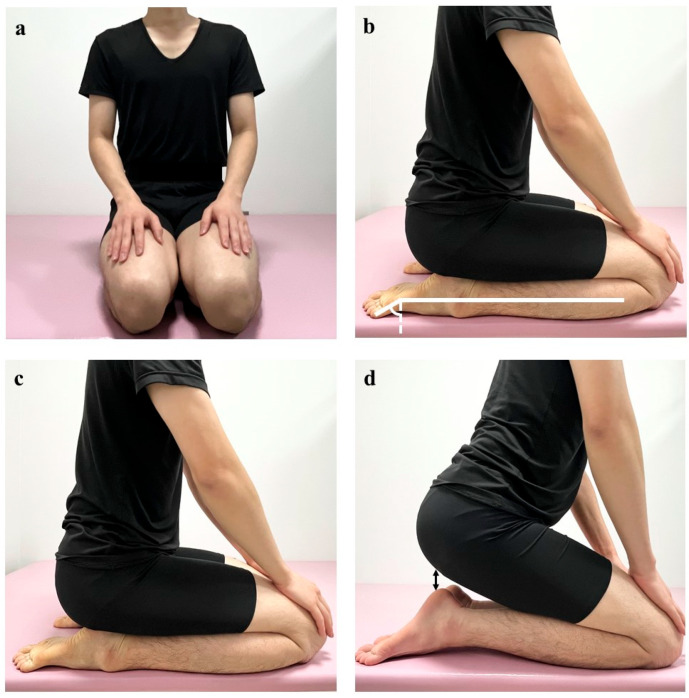
Seiza posture. (**a**) Anterior view of the seiza position. (**b**) Measurement of ankle plantarflexion angle during seiza. (**c**) Lateral view without seiza limitation. The hip, knee, and ankle joints exhibit adequate flexion, allowing the buttocks to rest fully on the heels. (**d**) Lateral view with seiza limitation. Reduced ankle plantarflexion prevents the buttocks from contacting the heels.

The seiza posture involves kneeling with the ankles fully plantarflexed and the buttocks resting on the heels [1] (Figure 2). Measurements were conducted by a physical therapist with 9 years of experience to ensure reliability. This position was held for 5 s to measure the ankle plantarflexion angle using a goniometer, defined as the angle between the plantar surface of the foot and a line connecting the fibular head and the lateral malleolus (Figure 2), with a minimum measurable value of 1°. To assess intrarater reliability, the same examiner repeated the measurement immediately after the initial measurement.

Seiza ability was assessed by measuring the heel–buttock distance in the seiza position. Seiza limitation was defined as a heel–buttock distance > 0 cm in the seiza position. Patients were categorised into two groups based on heel–buttock distance: a seiza-restricted group (>0 cm) and a non-seiza-restricted group (0 cm). This classification was used for subsequent analysis of factors related to seiza ability.

### 2.3. Measurement of the Shear Modulus and Architecture of the TA Muscle

The shear modulus of the TA muscle was measured using SWE in the Opt penetration mode with a 2 to 10 MHz linear transducer (Supersonic Imaging, Aix-en-Provence, France). The shear modulus reflects a muscle’s resistance to shear deformation. It is influenced by passive tissue properties and active muscle tone. Thus, SWE provides valuable information on muscle stiffness and may indirectly indicate structural or functional changes. The SWE technique uses acoustic radiation force from the push pulse to generate shear waves within the tissue, and the shear modulus is calculated using the following equation [24]:

μ (kPa) = ρVs^2^

where μ is the shear modulus, ρ is the tissue density (assumed to be 1000 kg/m^3^ for muscle), and vs. is the shear wave velocity.

**Figure 3:**
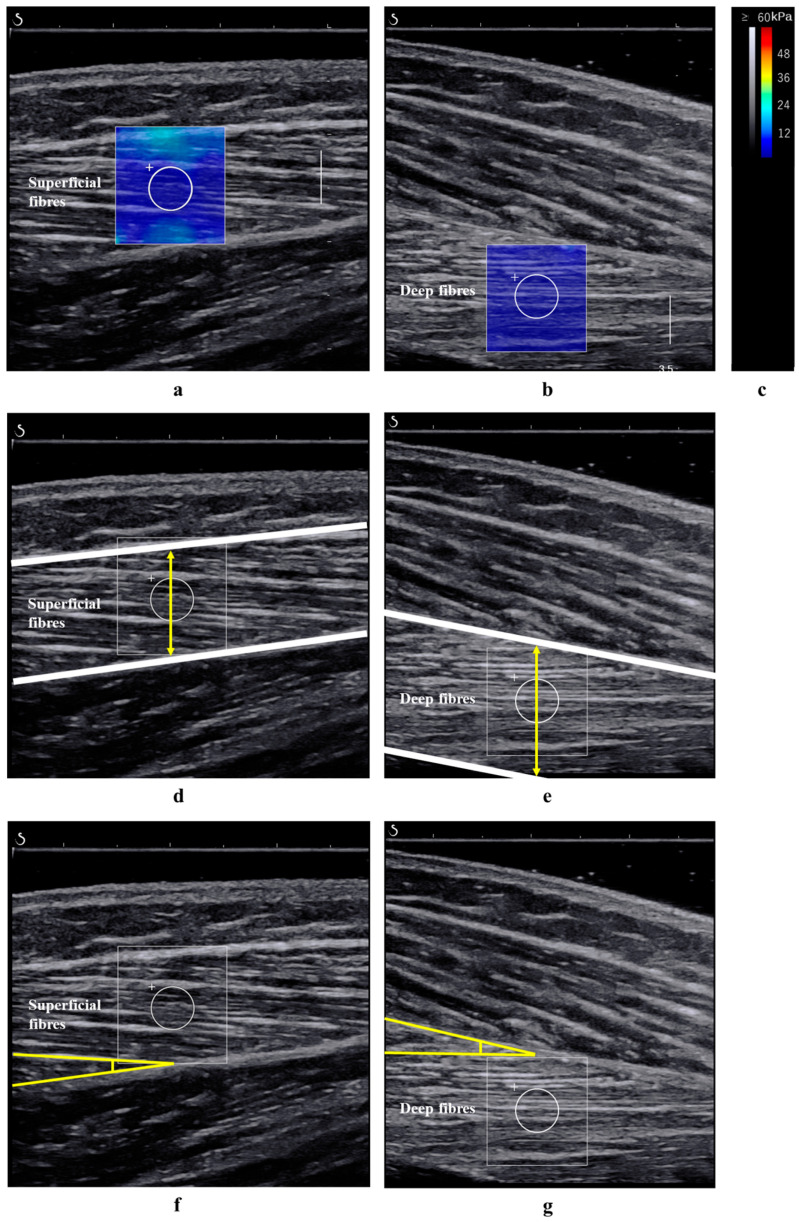
Shear modulus and architecture measurement. Shear modulus of the superficial (**a**) and deep (**b**) fibres of the TA muscle. (**c**) Colour scale. Muscle thickness of the superficial (**d**) and deep (**e**) fibres of the TA muscle. Pennation angle of the superficial (**f**) and deep (**g**) fibres of the TA muscle. TA, tibialis anterior.

Ultrasound images were obtained by a physical therapist with 9 years of experience in musculoskeletal ultrasound. Before the measurements, the participants were seated at rest for 10 min. Measurements were then performed with the participants in the supine position with both knees fully extended and the lower limbs relaxed. The participants were verbally instructed to relax to avoid muscle contraction during the measurement. Additionally, participants were instructed to avoid any strenuous activity that could induce muscle soreness for 2 days prior to the measurement [25], and adherence was confirmed through self-reporting. To standardise the ankle positioning, a goniometer was used to set the ankle plantarflexion angle to 30°. The ankle was secured to the tilt-table footplate using a strap to maintain this angle. The measurement site for the TA muscle was defined at the proximal one-third level of the lower leg length, measured using a measuring tape from the lateral knee joint space to the lateral malleolus [26] and marked on the skin with a black pen by the same examiner to minimise interrater variability. This region was selected because it is considered to have a higher density of muscle fibres [26], which is thought to reflect changes in muscle stiffness more accurately. The shear modulus values were measured in the range of 0–60 kPa. The probe was aligned parallel to the orientation of both the superficial and deep fibres. For both the superficial and deep fibres of the TA muscle, the region of interest was set to a fixed diameter of 4 mm, as this represented the maximum size that could be consistently measured across all participants (Figure 3). The probe was held stationary for 10 s to ensure measurement stability. A sufficient amount of gel was applied, and the probe was placed without pressure to ensure optimal shear wave propagation. The room temperature was maintained at 25 °C to prevent any changes in soft tissue stiffness due to temperature variations [27]. The average shear modulus value was calculated and used in the analyses. To avoid bias related to muscle activity, the examiner continuously monitored the TA using real-time B-mode imaging to confirm the absence of visible involuntary contractions. The thickness and pennation angle of the TA muscle were analysed using B-mode ultrasound images obtained during shear modulus measurements and ImageJ software version 1.54i (National Institutes of Health, Bethesda, MD, USA) (Figure 3). The thickness of the superficial and deep fibres of the TA muscle was measured as the distance from the superficial fascia to the intramuscular tendon and from the intramuscular tendon to the deep fascia, respectively [17]. The pennation angle of the superficial or deep fibres of the TA muscle was defined as the angle between the muscle fascicles and the intramuscular tendon [17]. The differentiation between the superficial and deep fibres of the TA muscle was achieved by identifying the intramuscular tendon within the muscle on ultrasound images. This tendon serves as a distinct anatomical landmark separating the two fibre layers. During shear wave elastography measurements, regions of interest were placed either superficial or deep to the intramuscular tendon to separately evaluate the mechanical properties of each layer. Measurements were repeated immediately to assess intrarater reliability.

### 2.4. Measurement of Ankle ROM

Ankle ROM was measured in 1° increments using a goniometer. The participants were placed in the supine position, and ankle dorsiflexion ROM was measured with the knee extended and flexed, and the plantarflexion ROM was measured with the knee flexed [28]. To measure passive ROM, the examiner passively dorsiflexed and plantarflexed the participant′s ankle to the maximum. For each measurement, the long axis was defined as the line perpendicular to the fibula, and the movement axis was aligned with the plantar surface of the foot. The angle formed between these two axes was recorded as the ankle dorsiflexion or plantarflexion ROM. The same examiner repeated measurements to assess intrarater reliability.

### 2.5. Measurement of Ankle Muscle Strength

Ankle plantarflexion and dorsiflexion muscle strengths were measured using a Biodex System 3 dynamometer (Biodex Medical Systems, Shirley, NY, USA). The participants were seated with their knees flexed at 30°, and their trunk, thighs, and ankles were secured using straps to minimise compensatory movements [28]. The axis of rotation of the dynamometer was aligned with that of the lateral malleolus. Isokinetic strength testing was performed bilaterally for plantarflexion and dorsiflexion using a concentric/concentric contraction mode. Two sets of five maximal dynamic repetitions were conducted at an angular velocity of 60°/s, with a 30 s rest between sets [28]. During each trial, the examiner provided verbal encouragement to ensure maximal effort. Peak torque values were recorded and normalised to the body weight (Nm/kg) for analysis. The highest peak torque value from all 10 repetitions was used for further analyses.

### 2.6. Measurement of Ankle Pain During Seiza

Ankle pain during seiza was self-reported by participants immediately after maintaining the seiza position for 5 s using a 100 mm visual analogue scale [29] under standardised conditions.

### 2.7. Statistical Analyses

The sample size was calculated using G*Power 3.1 (Heinrich Heine University, Düsseldorf, Germany) based on an effect size of f^2^ = 0.35 for multiple regression analysis, an alpha level of 0.05, and a statistical power of 0.80 [30], resulting in a minimum of 31 participants. A total of 38 participants were enrolled in this study. Normality was assessed with the Shapiro–Wilk test; normally distributed data are presented as mean ± standard deviation (SD), whereas non-normal data are presented as median (interquartile range). The reliability of the shear modulus and architecture of the TA muscle measurements, as well as the ankle plantarflexion angle during seiza and ankle ROM measurements, were evaluated using the intraclass correlation coefficient [ICC (1,1)] and Bland–Altman analysis based on the average of two measurements. Intrarater reliability was assessed using ICC (1,1), along with the standard error of measurement (SEM) [31], coefficient of variation in the differences (CV),95% confidence interval (CI) of the minimal detectable change (MDC) [32], and relative repeatability (RR) [33]. The strength of the reliability coefficient is defined as excellent (>0.90), good (0.71–0.90), moderate (0.50–0.70), and poor (<0.50) [34]. The formulae used were as follows:

SEM = SD × √(1 − ICC),

CV = (SD_diff_/(√2 × mean)) × 100,

MDC_95_ = 1.96 × SEM × √2, and

RR = MDC_95_/mean.

where SD_diff_ refers to the standard deviation of the differences between two repeated measurements.

Group comparisons (presence/absence of seiza restriction) were conducted using independent *t*-tests or Mann–Whitney U tests according to the data distribution. Sex distribution was compared using the chi-square test or Fisher’s exact test, depending on the expected frequencies. To examine associations between variables, Pearson’s product–moment correlation coefficients and Spearman’s rank correlation coefficients were calculated. Simple linear and stepwise multiple regression analyses identified the predictors of ankle plantarflexion angle during seiza, including TA shear modulus, age, BMI, number of fractures, ankle ROM, muscle strength, and pain. The Durbin–Watson ratio was used to test for autocorrelation in the residuals of the regression model, with values close to 2 indicating no autocorrelation. Additionally, multicollinearity among independent variables was assessed using the Variance Inflation Factor (VIF). A VIF value < 5 is generally considered acceptable and indicates that multicollinearity is not likely to distort the regression estimates [35]. These tests were conducted to ensure that the assumptions underlying multiple linear regression were adequately met, thereby supporting the validity of the analysis. The receiver operating characteristic (ROC) analysis was performed not to define the classification criteria, but to compare the discriminatory ability of the shear modulus values of the superficial and deep TA fibres in distinguishing between the predefined seiza-restricted and non-seiza-restricted groups. This analysis evaluated the discriminative performance of the TA shear modulus using the area under the curve (AUC), sensitivity, specificity, and optimal cut-off (Youden index) [36] to assess how accurately the TA shear modulus can identify the seiza restriction. AUC interpretation followed standard criteria: excellent, 0.90–1.00; good, 0.80–0.89; moderate, 0.70–0.79; poor, <0.70) [37]. In addition to the multiple linear regression and ROC analyses, we conducted a logistic regression analysis with presence/absence of seiza restriction as the dependent variable. Independent variables included the shear modulus of the superficial and deep TA fibres, and ankle pain during seiza. The model’s fit was assessed using the Hosmer–Lemeshow test, and multicollinearity was checked via variance inflation factors. Effect size for multiple regression was interpreted based on Cohen’s guidelines [38]: small (f^2^ = 0.02), medium (f^2^ = 0.15), and large (f^2^ = 0.35). The strength of the correlation coefficient was interpreted as follows: slight (<0.20), low (0.20–0.39), moderate (0.40–0.69), high (0.70–0.90), and very high (>0.90). For coefficient of determination (R^2^), values of 0.02, 0.13, and 0.26 were considered small, medium, and large, respectively.

Statistical significance was set at *p* < 0.05. All statistical analyses were performed using SPSS Statistics version 30 (IBM Corp., Armonk, NY, USA). There were no missing data for the analysed variables.

## 3. Results

### 3.1. Participants′ Characteristics

Among 49 patients with ankle fractures treated at our institution, 11 were excluded because of multiple fractures (n = 2), open fractures (n = 1), postoperative deep infection (n = 1), history of neurological disease (n = 1), refusal to undergo measurements (n = 2), and transfer to other hospitals (n = 4), resulting in a final sample of 38 participants (male, n = 18, 47.4%; female, n = 20, 52.6%; mean age, 45.8 ± 20.6 years). Of the 38 participants, 12 (heel–buttock distance > 0 cm) had limitations when assuming the seiza posture; 17 patients had unimalleolar fractures, 7 had bimalleolar fractures, and 14 had trimalleolar fractures. According to the Lauge–Hansen classification, 25 fractures were categorised as supination–external rotation, 4 as pronation–external rotation, and 9 as supination–adduction. The mean durations of immobilisation and load restriction following ORIF were 18.9 ± 11.7 and 49.5 ± 20.6 days, respectively. The mean time from ORIF to measurement was 91.8 ± 6.0 days.

### 3.2. Intrarater Reliability

**Figure 4:**
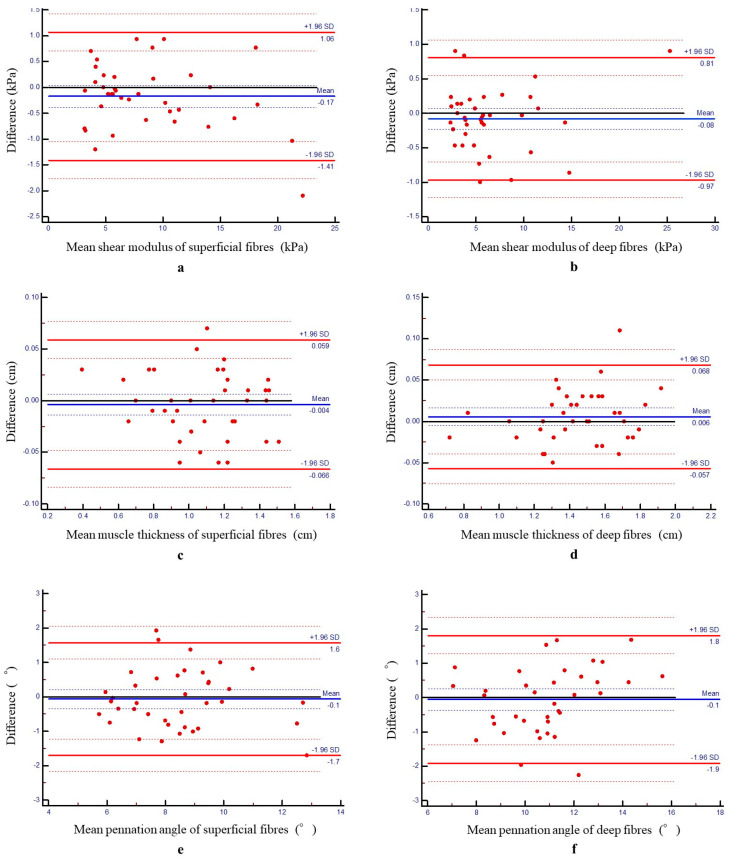
Bland–Altman plots of intrarater reliability of the shear modulus (**a**,**b**), muscle thickness (**c**,**d**), and pennation angle (**e**,**f**) of the TA muscle. Differences in shear modulus, muscle thickness, and pennation angle between the first and second measurements were plotted against the mean of each participant for the TA muscle. The blue line represents the mean of the differences, and the red line indicates the limits of agreement from −1.96 to +1.96 SDs. The blue and red dotted lines indicate the 95% confidence interval of the mean difference and the 95% confidence interval of ±1.96 SDs, respectively. TA, tibialis anterior; SD, standard deviation.

The intrarater reliability was excellent, with ICC values of 0.99 for shear modulus and muscle thickness, 0.89–0.90 for pennation angles, 0.99 for ankle plantarflexion angle during seiza, and 0.95–0.98 for ankle ROM (Table 1). No fixed or proportional bias was observed in the Bland–Altman analysis (Figure 4).

### 3.3. Comparison Between Two Groups Based on Seiza Ability (Seiza-Restricted Group vs. Non-Seiza-Restricted Group)

Table 2 shows a comparison of the participants′ characteristics and measurement variables according to the presence or absence of seiza limitation. The seiza-restricted group showed significantly higher shear modulus values in both the superficial (11.3 ± 6.9 vs. 7.3 ± 3.5 kPa, *p* = 0.008, r = 0.46) and deep fibres (11.1 ± 5.7 vs. 4.3 ± 1.2 kPa, *p* = 0.001, r = 0.77) of the TA muscle than the non-seiza-restricted group did. There were no significant differences between the groups in terms of age, sex distribution, or BMI.

### 3.4. Correlation Coefficients

The correlations between the ankle plantarflexion angle during seiza and other measurement values are shown in Table 3. The ankle plantarflexion angle during seiza correlated with the shear modulus of the deep (r = −0.731, *p* < 0.001) and superficial fibres (r = −0.692, *p* < 0.001), ankle plantarflexion ROM (r = 0.930, *p* < 0.001), and ankle pain during seiza (ρ = −0.585, *p* < 0.001). The other measurement parameters were not significantly correlated (*p* > 0.05).

### 3.5. Simple and Multiple Linear Regression Analyses

Simple linear regression analysis indicated that the shear modulus of the deep (β = −0.731, *p* < 0.001) and superficial fibres (β = −0.692, *p* < 0.001) and ankle pain during seiza (β = −0.585, *p* < 0.001) were significant predictors of the ankle plantarflexion angle during seiza. Multiple linear regression analysis further demonstrated that the shear modulus of the deep (β = −0.454, *p* < 0.001) and superficial fibres (β = −0.339, *p* = 0.017) were independent predictors of the ankle plantarflexion angle (Table 4). The Durbin–Watson ratio was 1.837, and the residuals showed no significant autocorrelation (*p* = 0.63).

### 3.6. ROC Analysis of Shear Modulus for Seiza Ability Prediction

**Figure 5:**
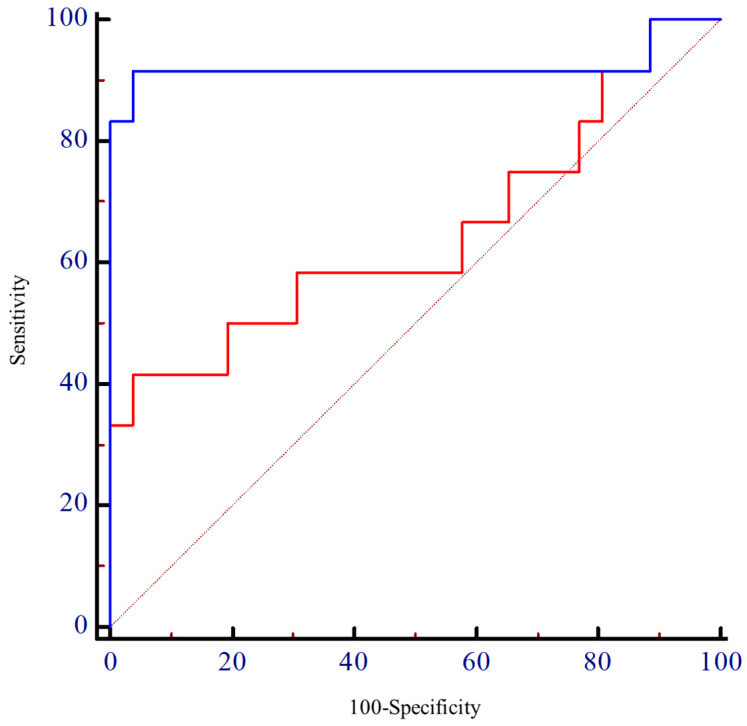
Receiver operating characteristic curve for the shear modulus of the TA muscle in discriminating seiza ability. The red and blue lines represent the superficial and deep fibres of the TA muscle, respectively. TA, tibialis anterior.

The ROC curve for the shear modulus of the deep fibres of the TA muscle (Figure 5) yielded an AUC of 0.92 (95% CI, 0.78–1.00). With a cutoff value of 6.0 kPa, the model achieved a sensitivity of 91.7% and a specificity of 96.2%. In contrast, the superficial fibres showed a lower discriminative ability, with an AUC of 0.65 (95% CI, 0.43–0.87). At a cutoff value of 8.7 kPa, the sensitivity and specificity were 41.7% and 96.2%, respectively.

### 3.7. Logistic Regression Analysis

Logistic regression analysis demonstrated that the shear modulus of the deep fibres of the TA muscle was significantly associated with seiza restriction (odds ratio = 2.72, 95% CI, 1.60–7.43, *p* = 0.006). In contrast, the shear modulus of the superficial fibres and ankle pain during seiza were not significant predictors after adjusting for the deep fibre modulus. The Hosmer–Lemeshow test indicated an acceptable model fit (*p* = 0.67), and the model correctly classified 94.7% of participants.

## 4. Discussion

In this study, we investigated the relationship between TA muscle stiffness and seiza ability after ankle fracture surgery. The main finding of this study was a significant association between increased shear modulus of the TA muscle and decreased ankle plantarflexion angle during the seiza posture. The shear modulus of the deep fibres of the TA muscle exhibited a strong negative correlation with the ankle plantarflexion angle (r = −0.73, *p* < 0.001), whereas the superficial fibres showed a moderate negative correlation (r = −0.69, *p* < 0.001). These results indicate that increased stiffness of the deep fibres is more strongly associated with limited ankle plantarflexion during seiza than stiffness of the superficial fibres did. To the best of our knowledge, this is the first study to identify the stiffness of the deep fibres of the TA muscle as an independent predictor of the ankle plantarflexion angle during seiza in patients after ankle fracture surgery.

The TA is the major dorsiflexor of the ankle and possesses a pennate structure [14]. Owing to their thicker architecture and greater pennation angle, the deep fibres may contribute more to passive resistance during plantarflexion than the superficial fibres [17]. Chleboun et al. [39] demonstrated an association between passive stiffness and muscle mass. The deep fibres of the TA muscle, owing to their proximal attachment near the tibia [40], are structurally positioned to respond directly to the mechanical tension exerted during plantarflexion and loading. In the traditional seiza posture, in which the ankle is placed under substantial plantarflexion and loading, these deep fibres are likely to be subjected to considerable tensile stress. Although a previous study has emphasised the general importance of dorsiflexor flexibility [41], our findings specifically highlight the regional contribution of the deep TA fibres. Although many studies have measured either the shear modulus of the superficial fibres of the TA muscle or that of the entire TA muscle [42,43,44], few have distinguished between and separately assessed the superficial and deep fibres of the TA muscle. This study adds to the current understanding by using SWE to quantify region-specific muscle stiffness, suggesting that deep fibre assessment may provide more clinically relevant insights than superficial measurements alone. These results support the need for targeted region-specific evaluations when assessing complex postures such as seiza [18,19]. In addition to the linear regression and ROC analyses, the inclusion of logistic regression further confirmed the predictive value of deep TA muscle stiffness for seiza restriction. This complementary analysis reinforced the clinical relevance of our findings by showing that deep fibres stiffness was an independent and significant predictor of functional limitation.

In addition, simple linear regression analysis revealed that ankle pain during seiza was significantly associated with the ankle plantarflexion angle in the seiza position. This pain may be influenced by the extensibility of the ankle dorsiflexion muscles. Muscle strength was not a significant predictor in our analysis. This may reflect the nature of the seiza posture, which relies more on passive flexibility [5,41] than on active force generation. During seiza, the ankle remains in a sustained, passively loaded position, requiring soft-tissue extensibility rather than muscular contraction.

Furthermore, we demonstrated good to excellent intrarater reliability of shear modulus measurements for the TA muscle in patients following ankle fracture surgery, based on the interpretation criteria proposed by Koo et al. [26]. Although Koo et al. [26] reported high reliability in healthy individuals, our findings extended these results to a postoperative population. The results of this study showed that the MDC_95_ for the shear moduli of the superficial and deep fibres of the TA muscle in participants after ankle fracture surgery were 0.9 and 1.2 kPa, respectively. The calculated MDC_95_ value provides a reference for distinguishing true changes from measurement variability, which may help clinicians and researchers track meaningful improvements over time. To evaluate whether the TA shear modulus could effectively distinguish between individuals with and without seiza restriction, we also conducted an ROC curve analysis. This approach allowed us to assess diagnostic accuracy by calculating the AUC, sensitivity, and specificity. The results revealed that the shear modulus of the deep fibres of the TA muscle demonstrated strong diagnostic performance, with an AUC of 0.92. At a cutoff point of 6.0 kPa, the model achieved a sensitivity of 91.7% and a specificity of 96.2%. In contrast, the superficial fibres showed a lower discriminative ability, with an AUC of 0.65, a sensitivity of 41.7%, and a specificity of 96.2% at a cutoff point of 8.7 kPa. Although the shear modulus of the superficial fibres was a significant predictor of the plantarflexion angle, the relatively low AUC may be due to the influence of confounding factors such as measurement variability, lesser involvement in passive resistance, or anatomical limitations.

Clinically, while these findings suggest a possible correlation between deep TA muscle stiffness and functional limitations, current evidence is insufficient to recommend specific interventions targeting deep TA fibres. Further research is necessary to evaluate the efficacy and safety of such approaches before they are applicable in clinical practice. 

This study has some limitations. First, muscle activity was not measured during the SWE assessment. Therefore, whether the increased stiffness reflects passive structural properties or a low-level involuntary contraction is unclear. However, to avoid bias related to muscle activity, the examiner continuously monitored the TA muscle using real-time B-mode imaging to confirm the absence of visible involuntary contractions. Second, muscle stiffness was assessed only at a single time point (3 months postoperatively), and longitudinal changes in TA elasticity were not evaluated. Additionally, the high reliability of the shear modulus measurements supports the use of SWE as a practical and objective tool for monitoring the progress of rehabilitation, although its applicability to other postoperative time points warrants further investigation. Moreover, although our cross-sectional design precluded repeated measures or bilateral analysis, future studies employing generalised linear mixed models could better account for both inter-limb and inter-individual variability. Third, the relatively small sample size, although based on power analysis, may limit the generalisability of our findings. Additionally, because the seiza posture is mainly practiced in Asian and Middle Eastern cultures, our results may not apply to populations where this posture is uncommon. Future studies with larger and geographically diverse samples are needed to confirm these findings and explore variability in TA stiffness and ability to assume the seiza posture. Finally, other potential contributors to seiza ability, such as joint capsule stiffness and subtalar joint involvement, were not assessed. Further research is required in this area.

## 5. Conclusions

Increased stiffness of the TA muscle, particularly in its deep fibres, is significantly associated with a reduced ankle plantarflexion angle during seiza in patients after ankle fracture surgery. These findings suggest that increased stiffness of the deep TA fibres may contribute to limitations in achieving the seiza posture. Given the clinical importance of this traditional position in the daily life of certain populations, region-specific evaluations and targeted interventions to address deep TA stiffness may improve functional recovery.

## Figures and Tables

**Table 1:** Intrarater reliability of ultrasound measurements of the TA muscle, ankle plantarflexion angle during seiza, and ankle ROM.

Measurement Variables	Test 1	Test 2	ICC	95% CI	SEM	CV (%)	MDC_95_	RR
Superficial fibres								
Shear modulus (kPa)	8.59	8.76	0.99	0.98–0.99	0.45	5.14	0.89	0.10
Muscle thickness (cm)	1.08	1.09	0.99	0.98–0.99	0.02	2.07	0.06	0.06
Pennation angle (°)	8.42	8.48	0.90	0.82–0.95	0.59	7.00	1.64	0.19
Deep fibres								
Shear modulus (kPa)	6.42	6.50	0.99	0.98–0.99	0.32	4.95	1.23	0.19
Muscle thickness (cm)	1.45	1.44	0.99	0.98–0.99	0.02	1.57	0.06	0.05
Pennation angle (°)	10.85	10.90	0.89	0.80–0.94	0.67	6.20	1.86	0.17
Plantarflexion angle during seiza (°)	57.9	57.7	0.99	0.98–0.99	0.59	1.02	1.64	0.03
Ankle ROM (°)								
Dorsiflexion with knee extended	12.7	12.6	0.95	0.91–0.98	0.54	4.27	1.50	0.12
Dorsiflexion with knee flexed	16.5	16.4	0.98	0.95–0.99	0.54	3.29	1.50	0.09
Plantarflexion	54.6	54.4	0.97	0.94–0.98	0.91	1.67	2.53	0.05

ICC, intraclass correlation coefficient; 95% CI, 95% confidence interval; SEM, standard error of the measurement; CV, coefficient of variation in the differences; MDC_95_, 95% confidence interval of the minimum detectable change; RR, relative repeatability; TA, tibialis anterior.

**Table 2:** Comparison of seiza ability between the seiza-restricted and non-seiza-restricted groups.

Variables	Seiza Ability		*p* Value	Effect Size
	Seiza-Restricted Group (n = 12)	Non-Seiza-Restricted Group (n = 26)		
Age (years)	46.8 ± 17.9	45.1 ± 22.7	0.819	0.04
Sex (male/female)	4/8	14/12	0.239	0.19
Height (m)	1.58 ± 0.10	1.62 ± 0.07	0.121	0.26
Weight (kg)	62.3 (56.0–71.3)	58.0 (50.5–71.5)	0.471	0.12
Body mass index (kg/m^2^)	26.0 ± 4.8	23.6 ± 4.9	0.176	0.22
Muscle thickness (cm)				
Superficial fibres	1.06 ± 0.18	1.09 ± 0.30	0.684	0.07
Deep fibres	1.39 ± 0.28	1.47 ± 0.25	0.373	0.15
Pennation angle (°)				
Superficial fibres	8.8 ± 2.3	8.2 ± 1.6	0.360	0.15
Deep fibres	10.5 ± 2.2	11.0 ± 2.1	0.510	0.11
Shear modulus (kPa)				
Superficial fibres	11.3 ± 6.9	7.3 ± 3.5	0.008	0.46
Deep fibres	11.1 ± 5.7	4.3 ± 1.2	0.001	0.77
Dorsiflexion ROM with knee extended (°)	14.0 (11.8–14.3)	12.0 (10.0–14.0)	0.211	0.20
Dorsiflexion ROM with knee flexed (°)	15.5 (14.0–18.0)	17.5 (15.0–19.3)	0.527	0.10
Plantarflexion ROM (°)	50.8 ± 6.3	56.3 ± 3.0	0.012	0.62
Plantarflexion angle during seiza (°)	54.3 ± 6.8	59.5 ± 3.5	0.012	0.40
Plantarflexion muscle strength (Nm/kg)	0.30 ± 0.18	0.39 ± 0.20	0.185	0.22
Dorsiflexion muscle strength (Nm/kg)	0.31 (0.25–0.34)	0.36 (0.27–0.43)	0.123	0.25
Ankle pain during seiza (mm)	30.0 (20.0–50.0)	0.0 (0.0–0.0)	0.013	0.40

Groups were defined based on participants′ clinical ability to assume the seiza posture at 3 months postoperatively. Seiza-restricted: unable to assume the seiza posture; non-seiza-restricted: able to assume the seiza posture. ROM, range of motion.

**Table 3:** Correlation coefficients between the ankle plantarflexion angle during seiza and other measurement variables.

Variables	Ankle Plantarflexion Angle During Seiza	
	Correlation Coefficient	*p* Value
Muscle thickness		
Superficial fibres	0.216 ^a^	0.193
Deep fibres	0.161 ^a^	0.335
Pennation angle		
Superficial fibres	−0.272 ^a^	0.100
Deep fibres	0.204 ^a^	0.219
Shear modulus		
Superficial fibres	−0.692 ^a^	<0.001
Deep fibres	−0.731 ^a^	<0.001
Ankle range of motion		
Dorsiflexion with extended knee	0.172 ^b^	0.303
Dorsiflexion with flexed knee	0.298 ^b^	0.069
Plantar flexion	0.930 ^a^	<0.001
Ankle strength		
Plantarflexion	0.225 ^a^	0.175
Dorsiflexion	0.243 ^b^	0.141
Ankle pain during seiza	−0.585 ^b^	<0.001

^a^  Pearson′s product moment correlation coefficient; ^b^  Spearman′s rank correlation coefficients.

**Table 4:** Simple and multiple linear regression analyses.

Dependent Variables	Simple Linear Regression		Multiple Linear Regression			
	β	*p* Value	β	*p* Value	R^2^	VIF
Shear modulus deep fibres	−0.731	<0.001	−0.454	<0.001	0.624	1.651
Shear modulus superficial fibres	−0.692	<0.001	−0.339	0.017		1.801
Ankle pain during seiza	−0.585	<0.001	−0.151	0.249		1.631

β, standardised regression coefficient; R^2^, adjusted coefficients of determination; VIF, variance inflation factor.

## Data Availability

The data supporting the findings of this study are available upon request from the corresponding author. The data are not publicly available owing to restrictions on their containing information that could compromise the privacy of the research participants.

## Abbreviations

The following abbreviations are used in this manuscript:

AUC	Area under the curve
CI	Confidence interval
CV	Coefficient of variation in the differences
ICC(1,1)	Intraclass correlation coefficient
MDC	Minimal detectable change
ORIF	Open reduction and internal fixation
ROC	Receiver operating characteristic
ROM	Range of motion
RR	Relative repeatability
SEM	Standard error of measurement
SWE	Shear wave elastography
TA	Tibialis anterior

## References

[1] Demura S., Uchiyama M. (2005). Effect of Japanese sitting style (seiza) on the center of foot pressure after standing. J. Physiol. Anthr. Appl. Hum. Sci..

[2] Fujita K., Makimoto K., Mawatari M. (2016). Three-year follow-up study of health related QOL and lifestyle indicators for Japanese patients after total hip arthroplasty. J. Orthop. Sci..

[3] Moon M.S., Lee H., Kim S.T., Kim S.J., Kim M.S., Kim D.S. (2018). Spinopelvic orientation on radiographs in various body postures: Upright standing, chair sitting, Japanese style kneel sitting, and Korean style cross-legged sitting. Clin. Orthop. Surg..

[4] Niki Y., Takeda Y., Harato K., Suda Y. (2015). Factors affecting the achievement of Japanese-style deep knee flexion after total knee arthroplasty using posterior-stabilized prosthesis with high-flex knee design. J. Orthop. Sci..

[5] Niki Y., Takeda Y., Udagawa K., Enomoto H., Toyama Y., Suda Y. (2013). Is greater than 145 degrees of deep knee flexion underweight bearing conditions safe after total knee arthroplasty?: A fluoroscopic analysis of Japanese-style deep knee flexion. Bone Jt. J..

[6] Elsoe R., Ostgaard S.E., Larsen P. (2018). Population-based epidemiology of 9767 ankle fractures. Foot Ankle Surg..

[7] Ohno H., Murata M., Ozu S., Matsuoka N., Kawamura H., Iida H. (2016). Midterm outcomes of high-flexion total knee arthroplasty on Japanese lifestyle. Acta Orthop. Traumatol. Turc..

[8] Kitagawa T., Nakase J., Takata Y., Shimozaki K., Asai K., Toyooka K., Tsuchiya H. (2019). Relationship between the deep flexion of the knee joint and the dynamics of the infrapatellar fat pad after anterior cruciate ligament reconstruction via ultrasonography. J. Phys. Ther. Sci..

[9] Torii Y., Yagi K., Izumi K., Jones B. (2019). The beneficial effect of mobilisation with movement therapy for patients with limited plantar flexion after malleolar fracture surgery: A case study. J. Man. Phys. Ther..

[10] Court-Brown C.M., Caesar B. (2006). Epidemiology of adult fractures: A review. Injury.

[11] Thur C.K., Edgren G., Jansson K.Å., Wretenberg P. (2012). Epidemiology of adult ankle fractures in Sweden between 1987 and 2004: A population-based study of 91,410 Swedish inpatients. Acta Orthop..

[12] Kannus P., Palvanen M., Niemi S., Parkkari J., Järvinen M. (2008). Stabilizing incidence of low-trauma ankle fractures in elderly people Finnish statistics in 1970–2006 and prediction for the future. Bone.

[13] Ekinci M., Birisik F., Ersin M., Şahinkaya T., Öztürk İ. (2021). A prospective evaluation of strength and endurance of ankle dorsiflexors-plantar flexors after conservative management of lateral malleolar fractures. Turk. J. Phys. Med. Rehabil..

[14] Keith L., Moore A., Agur A. (2006). Clinically Oriented Anatomy.

[15] Maganaris C.N., Baltzopoulos V. (1999). Predictability of in vivo changes in pennation angle of human tibialis anterior muscle from rest to maximum isometric dorsiflexion. Eur. J. Appl. Physiol. Occup. Physiol..

[16] Alexander R.M. (1975). The dimensions of knee and ankle muscles and the forces they exert. J. Hum. Mov. Stud..

[17] Martin-Rodriguez S., Gonzalez-Henriquez J.J., Galvan-Alvarez V., Cruz-Ramírez S., Calbet J.A., Sanchis-Moysi J. (2023). Architectural anatomy of the human tibialis anterior presents morphological asymmetries between superficial and deep unipennate regions. J. Anat..

[18] Miyasaka H., Ebihara B., Fukaya T., Mutsuzaki H. (2024). Absolute reliability of Young’s modulus of the soleus muscle and Achilles tendon measured using shear wave elastography in healthy young males. Asia Pac. J. Sports Med. Arthrosc. Rehabil. Technol..

[19] Koo T.K., Guo J.Y., Cohen J.H., Parker K.J. (2013). Relationship between shear elastic modulus and passive muscle force: An ex-vivo study. J. Biomech..

[20] Raiteri B.J., Cresswell A.G., Lichtwark G.A. (2018). Muscle-tendon length and force affect human tibialis anterior central aponeurosis stiffness in vivo. Proc. Natl. Acad. Sci. USA.

[21] Fonseca L.L.D., Nunes I.G., Nogueira R.R., Martins G.E.V., Mesencio A.C., Kobata S.I. (2018). Reproducibility of the Lauge-Hansen, Danis-Weber, and AO classifications for ankle fractures. Rev. Bras. Ortop..

[22] Budny A.M., Young B.A. (2008). Analysis of radiographic classifications for rotational ankle fractures. Clin. Podiatr. Med. Surg..

[23] Lauge-Hansen N. (1949). Ligamentous ankle fractures; diagnosis and treatment. Acta Chir. Scand..

[24] Gennisson J.L., Deffieux T., Macé E., Montaldo G., Fink M., Tanter M. (2010). Viscoelastic and anisotropic mechanical properties of in vivo muscle tissue assessed by supersonic shear imaging. Ultrasound Med. Biol..

[25] Muanjai P., Mickevicius M., Snieckus A., Jones D.A., Zachovajevas P., Satkunskiene D., Venckunas T., Kamandulis S. (2021). Response of knee extensor muscle-tendon unit stiffness to unaccustomed and repeated high-volume eccentric exercise. Int. J. Environ. Res. Public Health.

[26] Koo T.K., Guo J.Y., Cohen J.H., Parker K.J. (2014). Quantifying the passive stretching response of human tibialis anterior muscle using shear wave elastography. Clin. Biomech..

[27] Kot B.C.W., Zhang Z.J., Lee A.W.C., Leung V.Y.F., Fu S.N. (2012). Elastic modulus of muscle and tendon with shear wave ultrasound elastography: Variations with different technical settings. PLoS ONE.

[28] Miyasaka H., Ebihara B., Fukaya T., Mutsuzaki H. (2024). Evaluation of the relationship between echo intensity and Young’s modulus of the soleus muscle using ultrasound images after ankle fracture surgery. Cureus.

[29] Joyce C.R., Zutshi D.W., Hrubes V., Mason R.M. (1975). Comparison of fixed interval and visual analogue scales for rating chronic pain. Eur. J. Clin. Pharmacol..

[30] Faul F., Erdfelder E., Buchner A., Lang A.-G. (2009). Statistical power analyses using G*Power 3.1: Tests for correlation and regression analyses. Behav. Res. Methods.

[31] Weir J.P. (2005). Quantifying test–retest reliability using the intraclass correlation coefficient and the SEM. J. Strength. Cond. Res..

[32] Faber M.J., Bosscher R.J., Van Wieringen P.C.W. (2006). Clinimetric properties of the performance-oriented mobility assessment. Phys. Ther..

[33] Shankar H., Taranath D., Santhirathelagan C.T., Pesudovs K. (2008). Anterior segment biometry with the Pentacam: Comprehensive assessment of repeatability of automated measurements. J. Cataract. Refract. Surg..

[34] Domholdt E. (1993). Physical Therapy Research: Principles and Applications.

[35] James G., Witten D., Hastie T., Tibshirani R. (2013). An Introduction to Statistical Learning: With Applications in R.

[36] Akobeng A.K. (2007). Understanding diagnostic tests 3: Receiver operating characteristic curves. Acta Paediatr..

[37] Yatsuya H., Li Y., Hirakawa Y., Ota A., Matsunaga M., Haregot H.E., Chiang C., Zhang Y., Tamakoshi K., Toyoshima H. (2018). A point system for predicting 10-year risk of developing type 2 diabetes mellitus in Japanese men: Aichi workers’ cohort study. J. Epidemiol..

[38] Cohen J. (1988). Statistical Power Analysis for the Behavioral Sciences.

[39] Chleboun G.S., Howell J.N., Conatser R.R., Giesey J.J. (1997). The relationship between elbow flexor volume and angular stiffness at the elbow. Clin. Biomech..

[40] Kimata K., Otsuka S., Yokota H., Shan X., Hatayama N., Naito M. (2022). Relationship between attachment site of tibialis anterior muscle and shape of tibia: Anatomical study of cadavers. J. Foot Ankle Res..

[41] McNeill W. (2017). Adapting to floor sitting and kneeling. J. Bodyw. Mov. Ther..

[42] Souron R., Bordat F., Farabet A., Belli A., Feasson L., Nordez A., Lapole T. (2016). Sex differences in active tibialis anterior stiffness evaluated using supersonic shear imaging. J. Biomech..

[43] Boulard C., Mathevon L., Arnaudeau L.F., Gautheron V., Calmels P. (2021). Reliability of shear wave elastography and ultrasound measurement in children with unilateral spastic cerebral palsy. Ultrasound Med. Biol..

[44] Cornelson S.M., Ruff A.N., Perillat M., Kettner N.W. (2021). Sonoelastography of the trunk and lower extremity muscles in a case of Duchenne muscular dystrophy. J. Ultrasound.

